# Measurement and accountability for maternal, newborn and child health: fit for 2030?

**DOI:** 10.1136/bmjgh-2020-002697

**Published:** 2020-07-05

**Authors:** Tanya Marchant, Ties Boerma, Theresa Diaz, Luis Huicho, Catherine Kyobutungi, Claire-Helene Mershon, Joanna Schellenberg, Kate Somers, Peter Waiswa, On behalf of the Nairobi Group, Ambrose Agweyu

**Affiliations:** 1 Disease Control, London School of Hygiene & Tropical Medicine, London, UK; 2 Community Health Sciences, University of Manitoba, Winnipeg, Manitoba, Canada; 3 Department of Maternal, Newborn, Child, Adolescent Health and Aging, World Health Organization, Geneve, Switzerland; 4 Universidad Peruana Cayetano Heredia, Lima, Peru; 5 African Population and Health Research Center, Nairobi, Kenya; 6 Bill & Melinda Gates Foundation, Seattle, Washington, USA; 7 School of Public Health, Makerere University, Kampala, Uganda

**Keywords:** maternal health, child health, health systems evaluation

**Summary box**

- At the onset of the Sustainable Development Goals, in 2015, a group of global health experts delivered a call to action for an improved measurement system for women’s and children’s health.
- Five principles were defined, including having a focused set of core indicators, making data relevant to countries, investing in innovations, embedding equity measures and supportive global leadership.
- Five years later, in 2020, a second meeting reviewed progress against these principles and identified gaps and opportunities for investment in the coming decade.
- The greatest opportunity now is to make an intentional shift from global to local actions that strengthen measurement systems in the locations where they are needed.
- Greater country ownership of the measurement and accountability agenda is needed to promote more context-specific actions that reflect multisectoral realities, and that are supported by a responsive and adaptable measurement community.

## Introduction

As the current global COVID-19 pandemic makes clear, data are power. Now more than ever it is important to reflect on who holds that power and how well it is used to improve global health. This was also on the minds of a group of global health experts at the onset of the Sustainable Development Goals (SDGs). In 2015, after a first meeting in Kirkland, USA, these experts delivered a call to action for a robust maternal, newborn and child health (MNCH) measurement system that could effectively measure and monitor the coverage of high-impact healthcare while also improving capacity to track universal health coverage for women and children.^1^ That call to action defined five principles. There should be (1) a core focus on a set of indicators; (2) data relevant to countries; (3) measurement innovations; (4) embedded equity analysis and (5) global leadership.

Five years later, in 2020, MNCH measurement experts reconvened in Nairobi, Kenya, to reflect on progress against these principles and identify successes, gaps and opportunities. The most important required change is a shift from global to local actions that aim to strengthen measurement systems in the locations where they are most needed. Reflecting this, the Nairobi group added country ownership as a sixth measurement principle. Promoting the need for more context-specific actions that reflect multisectoral realities, and that are supported by a responsive and adaptable measurement community.

## The principle of focus

The principle of focus stated the need for a core set of global indicators of effective interventions with targets and measurement methods tailored to local settings. A set of harmonised tracer indicators to underpin global accountability mechanisms continues to be crucial. Going forward, countries need their own focused sets of measures, with greater attention given to the data required for decision making at different levels of the health system.

Globally, much progress has been made to identify a core indicator set for the SDG era.^2^ Continuing to limit this to an idealised small number is challenging, but this is not surprising given that SDG targets now range across sexual, reproductive, maternal, newborn, child and adolescent health plus nutrition. Or, for example, the recognition that stillbirths must be measured despite the lack of an SDG target. Added to this is the imperative to also measure the quality of care provided to populations. On measurement methods, academics have developed and tested improvements, United Nations agencies have reviewed best practices and invested in producing guidance, international survey programmes have revised their instruments, and more harmonised, country-level coverage data are now freely available online.^3^ Compared with 2015, the ability in 2020 to track priority indicators in a harmonised way is vastly improved.

But progress on focus is less clear when framed from a country perspective. Why might this be?

The majority of countries do now have a set of standards from which to build robust measurement plans, but with guidance for new measurements added without equal guidance provided on how to manage the growing reporting burden. The international periodic surveys that deliver on a core set of focused indicators are embedded in planning cycles in many countries, but they do not provide the real-time data needed for monitoring and course correction. More and more health facility and health system data are collected, which in turn places a heavy burden on health staff. Added to this mix is the global push to use modelled estimates (predictions) for programme monitoring when real data in real time is better fit for purpose.^4^ The various data platforms need to be rationalised, core indicator sets contextualised, and the culture of data use for decision making strengthened at different levels of health systems.

## The principle of relevance

The principle of relevance was about making MNCH coverage data useful in the country where it is collected. While reaffirming this principle, many gaps were observed. Going forward, relevance must be defined by context. Support to country institutions and people is needed, so that each setting can lead in defining, generating and using relevant data for decision making.

A gap is apparent between improvements in data availability and accessibility relative to the extent data are used for decision making in countries. What might explain this? In part, having more and more data to manage is time consuming and potentially overwhelming without sufficient resources. Further, often it is subnational-level decision making by health workers, by civil society and by government that can best drive programme improvement.^5^ But the technology that supports timely access to subnational data may be lacking. And subnational actors operate in a multisectoral system that tends to be data weak, potentially undermining a broad base culture of data use.

Realising the gains available from the proliferation of guidance needs actors at multiple levels to have the capacity, motivation, incentive and confidence to use data and not be overwhelmed by its complexity. Thus making data more relevant will need more investment in institutions and people. This area is a massive investment opportunity, maximised if those investments can be designed together with local institutions, include preservice training, have long-term vision and use adaptive management approaches.

## The principle of innovation

The principle of innovation was to develop efficient and technically sound methods and instruments, particularly for measuring the effective coverage of interventions. Progress has been made, but more consultation is needed, and there is a gap in the development of human-centred process innovations.

There have been a considerable number of innovations in the global and country tool kit for better measurement: digitisation, visual dashboards, league tables to name a few. Taking the example of effective coverage measurement, multiple dimensions of healthcare quality have been integrated in mainstream measurement guidance,^6^ digital tools have been developed, methods tested for linking relevant data sources and some engagement in countries undertaken to pressure test these innovations.

But to date there has been little evidence of translation within country plans. Why might this be? First, some of the innovations in methods and tools have emerged as a ‘push’ from global communities rather than a ‘pull’ from intended users. The underlying and often incorrect assumption being that if the right measurement innovations are made then there will be an inevitable trickle-down process from global to national plans. More effective interaction is needed. But second, there is an unmet need for process innovations that support people to use new methods and tools. Innovations that are designed to strengthen capabilities in data interpretation, innovations that make clear how a change will lead to an improvement, innovations that improve efficiencies for front-line health workers. And finally, new tools need to fit to the available technology platforms and currently these differ dramatically between countries.

## The principle of equity

The principle of equity was that equity must be a fundamental, rights-based component of programme design, measurement and management to direct resources to those most in need. Inequities have become highly visible in the SDG era. Considerable progress has been made in the technical work of inequality measurement and a plethora of disaggregated coverage and outcome data exists. Going forward, multisectoral leadership is needed to illuminate and take action on the underlying factors that persistently drive inequalities in health.

On the whole, the coverage of healthcare for women and children is increasing and disparities are narrowing in many settings.^7^ But despite wide-spread description, the same types of inequalities often persist. Why might this be? Partly due to a capacity gap, there is a disconnect between good situation analyses and appropriate programme planning that aligns with identified problems. And this may be exacerbated because the units of disaggregation can appear to be more academic than actionable. For example, categories of relative wealth, education and gender do not easily translate into action plans at local levels, since governments tend to allocate resources geographically, not to individual groups. Nonetheless, it is widely known that some population groups or some subnational areas are consistently left far behind their counterparts.^8^ Clearly, data alone are insufficient to stimulate action. The work to question why inequalities occur, to actively address and overcome their underlying determinants, is a complex task needing committed leadership that must extend beyond the health sector.

## The principle of leadership

The principle of leadership was to prioritise measurement and evaluation within the global agenda for MNCH and increase investment in data collection and use. Global leadership for women’s and children’s health continues to be critical in the crowded space of the multisectoral SDGs. But the opportunity now is to promote more participatory and distributive country-level leadership across different sectors.

Back in 2015 this principle was conceived to promote global level action by international funders and bilateral donors to invest in evidence and advocacy for women’s and children’s health, with a strong focus on equity. At that level, successful leadership that invested in and prioritised a better data system is evidenced through many large-scale and coordinated global efforts.^9^

In 2020, it is critical to consider what more can be done to support country-level leadership for measurement and accountability. Creating more actionable and relevant measurement systems requires participatory, distributed leadership, with technical capabilities developed across sectors, institutions and teams.^10^ Even though evidence is lacking on how to achieve this, investing in capacity within countries and promoting intersectionality will be key.^11^

## The principle of country ownership

Finally, 5 years after the first call to action, an additional principle of country ownership of the measurement and accountability agenda is conceived. This underpins the need to have focused and efficient measurement sets tailored at country level; relevant data that are defined according to individual contexts; innovations that invest in institutions and people; multisectoral partnerships to address inequalities; and leadership that is participatory and distributive.

## Conclusion

There is a lot to celebrate in the measurement of the expanded sector of sexual, reproductive, maternal, stillborn, newborn, child and adolescent health and nutrition. The sector leads the field of universal health coverage and effective coverage measurement and has numerous lessons to share. But to be fit for 2030, indeed to be ready to face new challenges today, the real test lies in the transition from global to local actions that strengthen measurement systems. A transition that will result in more countries gaining from the power of data and that will ultimately be reflected by greater country-owned accountability for health.
